# Lung on a Chip Development from Off-Stoichiometry Thiol–Ene Polymer

**DOI:** 10.3390/mi12050546

**Published:** 2021-05-11

**Authors:** Roberts Rimsa, Artis Galvanovskis, Janis Plume, Felikss Rumnieks, Karlis Grindulis, Gunita Paidere, Sintija Erentraute, Gatis Mozolevskis, Arturs Abols

**Affiliations:** 1 Institute of Solid-State Physics, University of Latvia, 8 Kengaraga Str., LV-1063 Riga, Latvia; roberts.rimsa@cfi.lu.lv (R.R.); karlis.grindulis@cfi.lu.lv (K.G.); gunita.paidere@cfi.lu.lv (G.P.); gatis.mozolevskis@cfi.lu.lv (G.M.); 2 Latvian Biomedical Research and Study Centre, Ratsupites Str 1, k-1, LV-1067 Riga, Latvia; artis.galvanovskis@biomed.lu.lv (A.G.); jan.jun.plume@gmail.com (J.P.); felikss.rumnieks@gmail.com (F.R.); sintija.erentraute@biomed.lu.lv (S.E.)

**Keywords:** lung on a chip, organ on a chip, off-stoichiometry thiol–ene, PDMS

## Abstract

Current in vitro models have significant limitations for new respiratory disease research and rapid drug repurposing. Lung on a chip (LOAC) technology offers a potential solution to these problems. However, these devices typically are fabricated from polydimethylsiloxane (PDMS), which has small hydrophobic molecule absorption, which hinders the application of this technology in drug repurposing for respiratory diseases. Off-stoichiometry thiol–ene (OSTE) is a promising alternative material class to PDMS. Therefore, this study aimed to test OSTE as an alternative material for LOAC prototype development and compare it to PDMS. We tested OSTE material for light transmission, small molecule absorption, inhibition of enzymatic reactions, membrane particle, and fluorescent dye absorption. Next, we microfabricated LOAC devices from PDMS and OSTE, functionalized with human umbilical vein endothelial cell (HUVEC) and A549 cell lines, and analyzed them with immunofluorescence. We demonstrated that compared to PDMS, OSTE has similar absorption of membrane particles and effect on enzymatic reactions, significantly lower small molecule absorption, and lower light transmission. Consequently, the immunofluorescence of OSTE LOAC was significantly impaired by OSTE optical properties. In conclusion, OSTE is a promising material for LOAC, but optical issues should be addressed in future LOAC prototypes to benefit from the material properties.

## 1. Introduction

There is currently evidence that the likelihood of pandemics will increase in the future because of increased global travel and integration, urbanization, changes in land use, and greater exploitation of the natural environment [1,2]. Respiratory diseases such as influenza, coronavirus, enterovirus, and other contagious respiratory pathogens are the main risks for potential pandemics [3]. In December 2019, pneumonia of an unknown etiology was confirmed in Wuhan, in the Chinese province of Hubei. The causal agent was soon identified as coronavirus, and the International Committee on Taxonomy of Viruses named the virus severe acute respiratory syndrome coronavirus (SARS-CoV-2). The World Health Organization (WHO) named the disease ‘2019-new coronavirus disease’ (COVID-19) and declared that COVID-19 was a fast-developing pandemic [1]. Globally, as of 9:36 am Central European Time (CET), 21 March 2021, there have been 122,271,944 confirmed cases of COVID-19, including 2,700,669 deaths, reported to WHO [4]. Currently, there are 5101 trials ongoing [5] for diagnostics, treatment, or prevention of COVID-19.

One of the most rapid ways to fight new pandemics is to repurpose existing drugs in the market. At the same time, it is also equally essential to increase the speed of new treatment development for different infectious respiratory diseases, including other coronaviruses, influenza, and enteroviruses. For this, in vitro models suitable for drug testing and recapitulating the behavior of different respiratory pathogens in the lungs are required. Currently, respiratory pathogens and drug efficacy in vitro are studied mainly by established cell lines, primary tissue-derived human cells, ex vivo human lung tissue cultures, and human lung organoids. However, all these in vitro models have significant limitations. For example, established cell lines often lack endogenous proteases necessary for viral propagation, and they lack highly differentiated tissue structures and functions seen in lungs, such as mucin layer formation [6]. Ex vivo cultures do not lack these characteristics; however, they have a limited cultivation time (usually just a few days), and they have limited reproducibility since they come from operation material [7]. Human lung organoids provide more differentiated tissue structures than cell cultures and longer incubation times than ex vivo tissue cultures. However, they lack air–liquid interface (ALI) and other physiologically relevant features of lungs, such as mucus layer formation and cocultivation of epithelium and endothelium cells.

Simultaneously, all these in vitro models are carried out under static conditions that poorly predict human responses to tested drugs’ pharmacokinetics [6,7]. Human lung on a chip (LOAC) technology offers a potential solution to these problems in an improved lung model system. The majority of LOAC devices consist of two microchannels separated by a porous membrane that allows cultivating lung epithelial cells on one side of the membrane and endothelial cells on the other, therefore mimicking epithelial–endothelial interface and air–liquid interface (ALI) of the human lung. Currently, there are multiple examples of LOAC devices that recapitulate human lung physiology and pathophysiology [8,9,10,11] better than static culture plates. As such, LOAC is a subset of lab-on-chip technology that deals with cell culturing on chips. Subsequently, it is no surprise that the majority of LOAC models are produced by using PDMS owing to the relatively cheap and simple fabrication process [12,13,14], gas permeability that simplifies device use in culture chambers [15,16], high transparency of material [17,18], and now an extensive database of multiple organ and organ system models published aiding with cell culture protocol development [19,20,21,22,23]. Furthermore, these LOAC devices’ membranes can also be made from PDMS, thus allowing a simplified device bonding strategy [24].

However, PDMS has several inherent issues limiting its application in LOAC development for drug development or repurposing of drugs. Of particular concern is the small, fat-soluble molecule absorption in PDMS [25,26]. One option to counter this is functionalizing the PDMS inner channels. Parylene [27], sol–gel-based silica nanoparticles [28], and Cellbinder [25] have been reported as promising coatings to reduce PDMS absorption of small, fat-soluble molecules. An alternative route to PDMS coating is elaborate modeling of the organ chip’s molecule diffusion, but this is especially computationally expensive when more than one organ model is connected [29]. Furthermore, the PDMS gas permeability is often seen as a negative feature of the material as it impedes the use of chips in normal culture chambers with altered gas contents, such as oxygen, to mimic hypoxia. Subsequently, it can be complex to reach physiologically relevant conditions in chips [30].

Off-stoichiometry thiol–ene (OSTE) is a promising alternative material class to PDMS, which was developed as an alternative to PDMS in the field of lab-on-chip. The use of Ultraviolet (UV)-curable off-stoichiometry thiol–enes offers multiple essential features due to the excess of thiol or epoxy groups during the curing stages. This article focuses on the OSTE materials developed by KTH (KTH Royal Institute of Technology, Stockholm, Sweden) and commercialized by Mercene Labs (Stockholm, Sweden), yet it is an active and exciting material class [31,32]. The required dual curing of the material (UV followed by thermal) allows retaining the ease of fabrication seen in PDMS devices without compromising resolution [33]. Excess of thiol groups on the surface allows for easy modification of the OSTE surfaces either after UV polymerization or after thermal curing steps via the thiol and hydroxy chemistries, respectively [34,35]. After thermal polymerization, OSTE becomes hard and obtains its final material properties. Of importance is the significantly lower gas permeability of OSTE; in comparison to PDMS, it is <10% of the water vapor value for the flexible OSTE material [36]. This can be especially useful in replicating hypoxic conditions in various organ-on-a-chip (OOC) devices, as PDMS chips suffer from oxygen ingress [15,30]. Finally, it previously has been shown that OSTE does not suffer from small molecule diffusion in the bulk of the material as PDMS does [26,36,37]. However, no LOAC devices from OSTE have been shown in the literature to the best of our knowledge. Therefore, this study aimed to test OSTE as an alternative material for LOAC prototype development, which could be applied in drug testing and repurposing for contagious respiratory diseases in the future. We demonstrated that OSTE has significantly less small molecule and fluorescent dye absorption than PDMS, as expected, while it has similar properties of membrane particle absorption and inhibitory effect on enzymatic reactions but lower light transmission than PDMS. We developed a simple LOAC microfluidic device from both OSTE and PDMS and compared functionalization with cell cultures based on immunofluorescence. Image analysis by confocal microscopy was significantly impaired due to the lower light transmission of OSTE, which affected the further development of LOAC from OSTE. In conclusion, LOAC from OSTE prototype optical properties should be addressed to make it applicable in researching respiratory diseases and drug development and repurposing in the future. 

## 2. Materials and Methods

### 2.1. PDMS and OSTE Test Piece Fabrication for Optical and Biocompatibility Property Evaluation

To directly evaluate and compare PDMS and OSTE material properties in enzymatic reactions, such as complementary DNA (cDNA) synthesis and Polymerase chain reaction (PCR), we fabricated ~1 mm thick round test pieces with 14 mm diameter. These test pieces were die-cut from large sheets of material using a manual die-cutter. PDMS sheet was fabricated by casting PDMS (Sylgard 184, Dow Corning, 1:10 crosslinker/base ratio, *w*/*w*) onto a glass plate with laser-cut cast acrylic sidewalls, degassing for 30 min in −800 mbar pressure, and curing at 85 °C in a convection oven (Shanghai Yuanhuai Industrial CO DZF-6020, Shanghai, China). After curing, the outer 2 mm of the material (i.e., closest to the mold’s acrylic sidewalls) was discarded to eliminate any PDMS material that could have been contaminated with acrylic leachates. OSTE sheets were fabricated by pouring OSTE 322 (Mercene Labs, mixed as per instructions on the bottle, 1.09:1 Part A/Part B, *w*/*w*) onto polystyrene Petri dishes with bottom surface roughness (R_a_) of around 20 nm, degassing for 30 min in –800 mbar pressure, and UV curing for 5 min with a UV intensity (i-line) of 6.8 mW/cm^2^. Finally, OSTE samples were cured on a hot plate at 60 °C overnight between two Polytetrafluoroethylene (PTFE) sheets to ensure OSTE did not stick to the hot plate surface.

### 2.2. PDMS and OSTE Comparison of Light Transmission

PDMS and OSTE sample optical properties were measured using a spectrophotometer (Cary7000, Agilent Technologies, Santa Clara, CA, USA) on previously described pieces. The spectra were measured in transmission mode for three samples from each material and averaged between 300 and 800 nm wavelengths with a 600 nm/min scan rate, an interval of 2 nm, and no light polarization. Measurements were performed twice for each sample. The sample transmission plots were obtained by integrating the total area under the curve and normalizing it to a soda-lime glass (Kyocera, Osaka, Japan) sample. Statistical significance was calculated by Mann–Whitney test.

### 2.3. PDMS and OSTE Comparison of Rhodamine Absorption

For evaluating the material absorption of small molecules, three pieces from each material were immersed in 100 µM rhodamine B solution, made by dissolving rhodamine B powder (Sigma-Aldrich, St. Louis, MO, USA) in de-ionized (DI) water for 24 h at a temperature of 20 °C. After immersion, samples were thoroughly rinsed with DI water before measuring transmission spectra. Measurements were performed twice. Sample absorption was evaluated as the integrated area of the trough between 450 and 600 nm. Area integration was done via Origin 9.0 software with a baseline fit to the curve to determine the trough. A soda-lime glass sample was used as a reference, which had no change in transmission spectra post rhodamine B immersion. The area under the curve results were normalized to the PDMS sample. Statistical significance was calculated by Mann–Whitney test.

### 2.4. PDMS and OSTE Polymer Effect on RNA Isolation, cDNA Synthesis, and Quantitative Reverse Transcription (qRT)-PCR Reaction

PDMS and OSTE round pieces were washed with 70% ethanol overnight, air dried, and washed with sterile 1× PBS overnight. Washed pieces were cultivated with 1 mL DMEM (41966-052, Thermo Fisher, Waltham, MA, USA) supplemented with 10% FBS (F7524-500 ML, Sigma-Aldrich) and 50 µL/mL Primocin (ant-pm-2, InvivoGen, San Diego, CA, USA) for 48 h in a humidified incubator at +37 °C, 5% CO_2_ to mimic cell culturing environment. After 48 h, 700 µL Qiazol from miRNeasy Micro Kit (217084, Qiagen, Hilden, Germany) was directly added in wells with polymers, representing cell lysing. Wells without polymers were used as controls. Experiments were performed in duplicates. One microliter (1 × 10^9^ copies/µL) of synthetic spike-in UniSp6 from miRCURY LNA RT Kit (339340, Qiagen) was added to the lysate to monitor polymer effect on RNA isolation, cDNA synthesis, and PCR reaction since it should be similar to the control sample. cDNA was synthesized by miRCURY LNA RT Kit, and qRT-PCR was performed by miRCURY LNA SYBR Green PCR Kits (339346, Qiagen) and UniSp6 primer assay (339306, Qiagen) by applying ViiA 7 Real-Time PCR System (Thermo Fisher). Reactions were performed in technical duplicates on each biological duplicate. Ct values were compared between samples, and the *p*-value was calculated by the Mann–Whitney test.

### 2.5. PDMS and OSTE Single-Channel Device Preparation

To test biological entities and in immunofluorescence applied dye absorption, PDMS and OSTE single-channel devices were microfabricated. PDMS devices were fabricated by casting PDMS (Sylgard 184, Dow Corning, Midland, MI, USA, 1:10 crosslinker/base ratio, *w*/*w*) onto custom machined aluminum molds and degassing around 30 min in −800 mbar pressure. The channel height was 180 µm, and channel width was 1 mm with surface roughness (R_a_) of around 30 nm parallel and orthogonal to the channels. After curing, devices were peeled out from the molds, and inlets were punched using a biopsy puncher. To bond the devices, PDMS and a clean soda-lime glass slide were placed in UV-Ozone cleaner (Novascan PSDP-UV8T, Boone, IA, USA) for 5 min; after ozone exposure, PDMS and glass were immediately brought into contact and cured overnight on a 65 °C hot plate with pressure on the stack of around 2 kPa.

OSTE devices were fabricated using OSTE 322 (Mercene Labs, Stockholm, Sweden), which was mixed according to the instructions (Part A to Part B 1.09:1 (*w*/*w*)) and degassed for 30 min at 800 mbar pressure shortly before casting onto the aluminum molds also used for PDMS device fabrication. OSTE was cured for 4 min with a UV intensity (i-line) of 6.8 mW/cm^2^. After curing, devices were peeled out from the molds, and inlets were punched using a revolving hole punch. Finally, the OSTE slabs were brought into contact with a clean soda-lime glass slide, and the whole assembly was cured on a 100 °C hot plate overnight with a pressure of around 8 kPa on the whole stack.

### 2.6. Membrane Particle Absorption of PDMS and OSTE

Extracellular vesicles (EVs) isolated from cell cultures were used to evaluate material properties on membrane particle absorption and mimic virus particles. EVs were isolated and characterized from ASC52-telo (SCRC-4000, ATCC, Manassas, VA, USA) cell culture similarly to previously described protocols [38,39]. Single-channel devices were washed with 70% ethanol overnight, dried, and washed with sterile 1× PBS overnight. Then, 20 µL of 1.02 × 10^8^ EVs/mL in 0.02 µm filtered 1× PBS were injected into channels and incubated for 1 h at +37 °C. After incubation, EV solutions from channels were collected and measured by nanoparticle tracking analysis (NTA) with an NS 300 instrument (Malvern, Philadelphia, PA, USA) equipped with green (532 nm) laser and scientific Complementary metal–oxide–semiconductor (sCMOS) camera and compared with input sample. Here, 0.02 µm filtered 1× PBS was used as a negative control. Measurements were performed on five 30 s videos that were recorded using camera level 12. The data were analyzed using NTA software v3.0 with the detection threshold 8 and screen gain at 5. Experiments were performed in biological duplicates and measured in technical duplicates. *p*-value was calculated by the Mann–Whitney test.

### 2.7. CellVue Absorption of PDMS and OSTE Materials

CellVue (MINCLARET-1KT, Sigma-Aldrich) was selected for dye absorption tests since it is a small fluorescent molecule often used in cell culture experiments to label cell membranes. Channels were washed with 20 µL Diluent C and incubated with 2 × 10^−6^ M CellVue dye in Diluent C according to the manufacturer’s protocol applied for cell membrane labeling and incubated for 1 h at +37 °C. Sterile 1× PBS was used as a control. Next, channels were washed 3 times with sterile 1× PBS and analyzed using a confocal laser scanning microscope (TCS SP8, Leica, Germany) with 633 nm excitation laser (helium-neon, Thorlabs Inc, Newton, NJ, USA). Image processing was done by ImageJ bundled with 64-bit Java 1.8.0_172 and used to quantify CellVue absorption. Normalized total fluorescence of CellVue was calculated by integrated density – (selected area × mean fluorescence of background), where integrated density summarizes all pixels in a selected area. Experiments were performed in biological duplicates, and all measurements were performed in technical duplicates. Statistical significance was calculated by Mann–Whitney test.

### 2.8. LOAC Device Microfabrication from PDMS and OSTE Polymers

PDMS devices were fabricated by casting PDMS (Sylgard 184, Dow Corning, 1:10 crosslinker/base ratio, *w*/*w*) onto custom machined aluminum molds and degassing of around 30 min in −800 mbar pressure. The top and bottom channels had a channel height of 200 µm; the top channel width was 1.2 mm, whereas the bottom channel width was 1.0 mm to account for alignment tolerances. Aluminum molds had a surface roughness (R_a_) of around 50 nm parallel and orthogonal to the channels. PDMS was cured for 2 h at 85 °C in a convection oven. Fluid outlets were then cut using a 1.25 mm biopsy puncher. 

**Figure 1:**
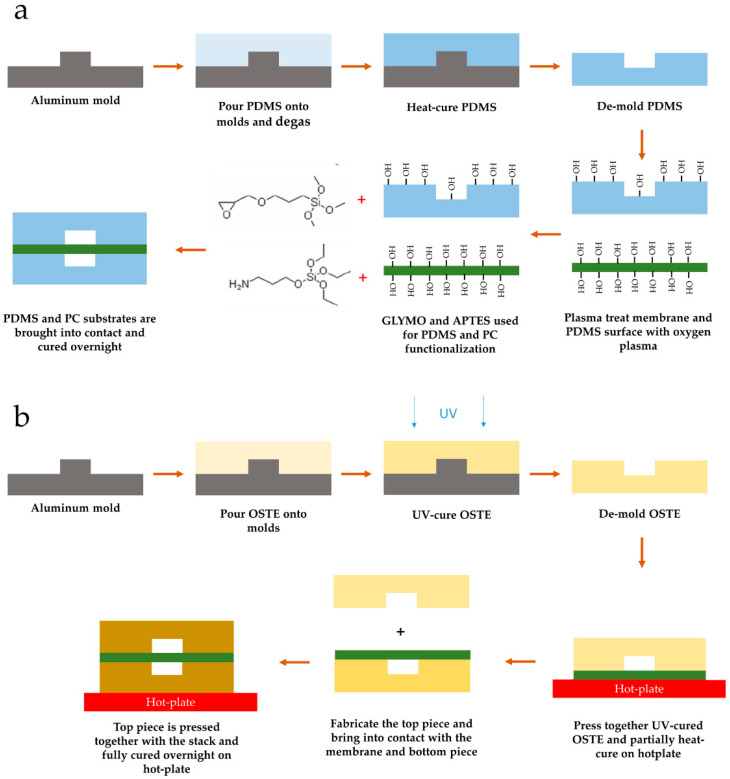
Lung on a chip (LOAC) device fabrication workflow. (**a**) Polydimethylsiloxane (PDMS) device fabrication workflow. The process is based on the epoxy–amine bonding process described previously [40]. Amine functionality was assigned to polycarbonate (PC) membrane to enhance extracellular matrix (ECM) adhesion to the membrane. (**b**) Off-stoichiometry thiol–ene (OSTE) device fabrication workflow. To retain membrane flatness during the assembly process, the bottom piece was pre-bonded and under-cured to the membrane first before bonding the top piece and fully curing the assembly.

PDMS channels were bonded to a track-etched polycarbonate (PC) membrane (it4ip S.A., Louvain-La-Neuve, Belgium) with a nominal pore size of 3 µm and density of 1.6 × 10^6^/cm^2^. Track-etched membranes were chosen due to their uniform pore distribution and highly controlled porosity. PC was the material of choice due to better bonding performance with OSTE materials, where generally polyethylene terephthalate (PET) is used as a release liner material. The bonding protocol consisted of a modified amine-epoxy protocol based on the previously described method [40]. PDMS surfaces were modified by immersion in 1% aqueous GLYMO (2530-83-8, Sigma-Aldrich) solution for 20 min after a 5 min oxygen plasma treatment step in a plasma asher (PVA TePla AG GIGAbatch 360M, Wettenberg, Germany); similarly, PC membranes were modified with 1% aqueous APTES (919-30-2, Sigma-Aldrich) solution for 20 min after a 1 min oxygen plasma treatment step. After surface modification, all three parts were blow-dried with nitrogen and immediately brought into contact using a custom jig. Further, the PDMS-PC-PDMS stack was cured overnight on a 65 °C hot plate with pressure on the stack of around 2 kPa as visualized in Figure 1a.

OSTE devices were fabricated using OSTE 322 (Mercene Labs), which was mixed according to the instructions (Part A to Part B 1.09:1 (*w*/*w*)) and degassed for 30 min at −800 mbar pressure shortly before casting onto the aluminum molds with a similar surface finish to PDMS devices. Top and bottom channels were made from the same aluminum molds with a channel height of around 180 µm and a width of 1.0 mm. The differences from PDMS molds stem from the tolerances in the computer numerical control (CNC) milling process. OSTE was filled into the mold cavity using a custom setup and cured for 4 min with a UV intensity (i-line) of 6.8 mW/cm^2^. After the UV curing step, the bottom pieces were pressed together with the same PC membrane and cured at 60 °C for 1 h on a hot plate with around 8 kPa pressure on the stack. The top piece was fabricated with the same parameters. After the UV curing step, inlets were punched using a revolving hole punch. The top piece was then brought into contact with the PC membrane, and the whole assembly was cured on a 60 °C hot plate overnight with a pressure of around 8 kPa on the whole stack, as visualized in Figure 1b. After bonding, devices were placed in a plasma asher (PVA TePla AG GIGAbatch 360M, Wettenberg, Germany) for 5 min to activate the PC membrane and OSTE surfaces. 

### 2.9. LOAC Device Preparation and Cell Cultivation

Before cell seeding, both PDMS and OSTE LOAC devices were washed in 70% ethanol overnight, dried, and washed in sterile 1× PBS overnight. Upper and lower channels were coated by 0.5 mg/mL collagen IV from human placenta (C5533-5MG, Sigma-Aldrich), diluted in sterile 1× PBS, and incubated overnight in cell incubator at +37 °C and 5% CO_2_. On the following day, 2E7 HUVEC-2 cells/mL (354151, Corning, NY, USA) were seeded in the bottom channel, and the device was inverted and left overnight in DMEM/F12 (31330095, Thermo Fisher) supplied with 1× LVES (A1460801, Thermo Fisher) and 50 µg/mL Primocin (ant-pm-2, InvivoGen) at +37 °C and 5% CO_2_ in static conditions for cell attachment. The next day, the LOAC device was inverted back, and A549 (CCL-185, ATCC, Manassas, VA, USA) cells were plated at a concentration of 3.5 × 10^6^ cells/mL in DMEM/F12 (31330095, Thermo Fisher) with 10% FBS (F7524-500ML, Sigma-Aldrich) and 50 µg/mL Primocin (ant-pm-2, InvivoGen) and left overnight for cells to attach at +37 °C and 5% CO_2_ in static conditions. Next, LOAC devices were connected to a syringe pump (ISPLab02, Baoding Shenchen Precision Pump Co. Ltd., Baoding, China), and top and bottom channels were perfused with their respective media at 2 µL/min per channel in the withdraw (vacuum) setting. Channel-specific media were preconditioned in the incubator for 1 h at +37 °C and 5% CO_2_ and stored inside the incubator during LOAC incubation. Media was changed every 48 h. After seven days, LOAC was analyzed by immunofluorescence and confocal microscopy to compare cell co-culture growing on both LOAC devices.

### 2.10. Immunofluorescence

Before immunofluorescence, primary antibodies were conjugated with either FITC Conjugation Kit (Fast) Lightning-Link (ab188285, Abcam, Cambridge, UK) or with Lightning-Link APC Antibody Labeling Kit (705-0030, Novus Biologicals, Littleton, CO, USA). Cells cultured in LOAC devices were washed by perfusing 1 mL of sterile 1× PBS; the membrane was labeled with CellVue Claret Far Red Fluorescent Cell Linker Mini Kit (MINCLARET, Sigma-Aldrich) by washing once with Diluent C, incubating with 2 × 10^−6^ M CellVue dye in Diluent C for 5 min, blocking the staining with 1% BSA (A7906, Sigma-Aldrich), and washing with 1 mL sterile 1× PBS. Cells were fixed with filtered 4% paraformaldehyde (P6148-500G, Sigma-Aldrich) solution in sterile 1× PBS for 30 min, washed with 1 mL sterile 1× PBS, and permeabilized with 0.5% Triton X-100 (93443, Sigma-Aldrich) for 5 min. Then, cells were blocked with 5% BSA (A7906, Sigma-Aldrich) for 2 h and washed with 1 mL sterile 1× PBS. Cells were then stained overnight at +4 C with FITC conjugated Anti-ZO1 tight junction protein antibody (ab216880, Abcam) diluted 1:100, FITC conjugated Anti-Mucin 5AC antibody (ab3649, Abcam) diluted 1:100, and APC conjugated Anti-CD31 antibody (ab9498, Abcam) diluted 1:500. After Ab incubation, channels were washed with 1 mL sterile 1× PBS, counterstained for 5 min with DAPI (D9542, Sigma-Aldrich), and washed with 1 mL PBS.

DAPI-stained channels of PDMS and OSTE chips were captured with EVOS M5000 imaging system (AMF5000, Invitrogen, Carlsbad, CA, USA). Channels were captured with transmitted light and EVOS Light Cube, DAPI 2.0 (AMEP4950, Invitrogen) using EVOS 10× Objective (AMEP4981, Invitrogen). Transmitted light and DAPI images were combined using EVOS M5000 built-in software to reflect cell distribution on the chip membrane.

### 2.11. Confocal Microscopy and Image Processing

Chips were imaged using a confocal laser scanning microscope (TCS SP8, Leica, Germany). Confocal Z-stacks were scanned using 10×/NA 0.30 objective (Leica, Germany). DAPI was imaged using a 405 nm excitation laser (diode), and FITC was imaged using a 488 nm excitation laser (argon). Cell Vue and APC were imaged using a 633 nm excitation laser (helium–neon). PDMS LOAC Z-stacks were scanned over a 150 μm range at 3 μm intervals to cover both HUVEC and A549 cell layers. OSTE LOAC images were captured at a single position on *Z*-axis. Images and Z-stack maximum projections were then processed using Leica Application Suite X software (Version 3.7.4.23463, Leica Microsystems, Germany). Three-dimensional Z-stack reconstruction was performed using LAS X 3-D Viewer (Leica Microsystems, Germany). 

## 3. Results

### 3.1. Comparison of OSTE and PDMS Characteristics Crucial for LOAC Development

**Figure 2:**
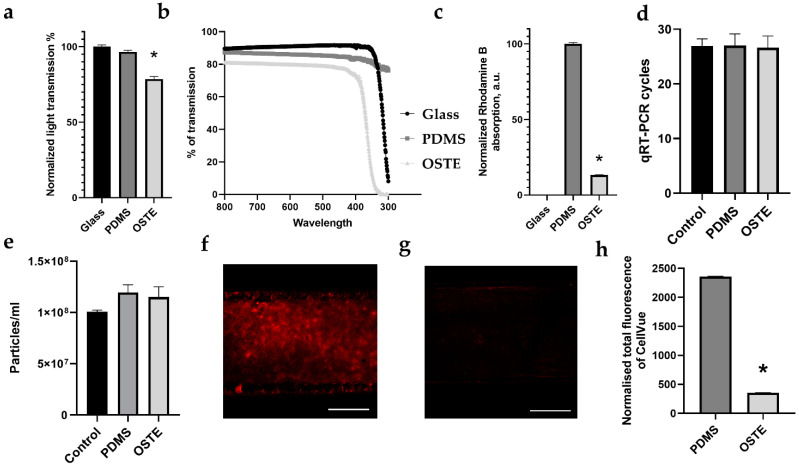
Comparison of PDMS and OSTE characteristics necessary for LOAC. (**a**) Comparison of total light transmission. (**b**) Comparison of light transmission across the 300–800 nm range. (**c**) Comparison of rhodamine absorption. (**d**) Comparison of PDMS and OSTE inhibition on quantitative reverse transcription (qRT)-PCR. Control represents lysate without polymer. (**e**) Comparison of extracellular vesicle absorption. Control represents input extracellular vesicle (EV) amount. (**f**) Image of CellVue absorption in PDMS single-channel device. (**g**) Image of CellVue absorption in OSTE single-channel device. (**h**) Quantitative comparison of CellVue absorption between PDMS and OSTE single channel devices. White scale bars represent 300 μm. Bars represent mean value, and whiskers represent standard error in (**a**,**b**,**c**,**h**) and standard deviation in (**d**,**e**). * represents *p* value below 0.05. A.u. stands for arbitrary numbers. Data analysis and preparation of graphs were performed with GraphPad Prism 8.4.3.

Crucial aspects of the LOAC and OOC overall in drug repurposing and development are cell monitoring by fluorescent or confocal microscopy, florescent dye, absorption of small molecules and larger entities, and effect on enzymatic reactions, which are usually applied in downstream analysis. PDMS and OSTE pieces were compared in terms of their relative light transmission across the 300–800 nm range, covering excitation and emission peaks for commonly used fluorescent dyes [41]. From Figure 2a, it can be seen that PDMS has almost the same light transmission as glass in the selected range. Normalized light transmission of glass is 100% standard error of the mean (SEM) ± 1.21% (the glass was used as reference), while PDMS has 96.62% SEM ± 1.01%, which has been long seen as one of the advantages of PDMS in optically sensitive applications [17,18]. In comparison to PDMS, OSTE has lower normalized light transmission—78.67% SEM ±1.65%. It has already been reported that OSTE 322 material seems to suffer from strong light scattering [32]. This likely explains the reduced transmission of the material across the whole investigated wavelength (300–800 nm) range seen in Figure 2b.

Small drug absorption by materials, which is crucial for drug studies, has [42] been characterized by rhodamine B absorption of PDMS and OSTE polymer pieces. Figure 2c shows normalized rhodamine B absorption that demonstrates that OSTE has significantly reduced small molecule absorption compared to PDMS, while glass has 0 absorptions compared to PDMS, which was used as reference (normalized rhodamine B absorption of glass 0%, SEM ±0%; PDMS 100%, SEM ±1%; OSTE 13.31%, SEM ±0.13%). These results confirm previously published results and suggest that OSTE is much better suited for LOAC microfabrication for drug testing purposes [43]. 

Next, given that there is a significant emphasis on cell analysis with PCR and sequencing in the OOC field, it is of high importance to evaluate if the materials utilized in LOAC devices would not affect any downstream enzymatic analysis methods. To evaluate this, PDMS and OSTE pieces were cultivated in cell culture media in the incubator to mimic cell culturing conditions and directly lysed with Qiazol to evaluate any leakage of material in the buffer during lysing that could hamper typical downstream cell analysis processes such as PCR. An equal amount of spike-in in PDMS, OSTE, and control lysates was added to quantify inhibition on qRT-PCR. Figure 2d shows qRT-PCR cycle threshold values for sample materials. Significant differences in cycle threshold values would show any leachates inhibiting the RNA isolation, cDNA synthesis, or qRT-PCR. However, there was no significant difference in spike-in amplification between control and PDMS samples, which is in line with previously published results [44]. At the same time, there were no significant differences between OSTE, PDMS, and control sample also, which suggests that OSTE is at least as good as PDMS for cell downstream analysis purposes.

To test PDMS and OSTE absorption of larger biological entities, we used single-channel PDMS and OSTE devices. Absorption of larger entities such as virus particles is crucial for research of respiratory disease by LOAC. We used extracellular vesicles (EVs) to mimic virus particles since they share some similarities with retroviruses, such as size and composition, and EVs often play significant roles in virus infection [45].

Figure 2e represents a number of particles in the input sample representing control and output sample from PDMS and OSTE channels measured by NTA. There was a slight increase in particle amount in both PDMS and OSTE samples compared to the control sample, 1.2 × 10^8^, standard deviation (SD) ± 8 × 10^6^; 1.15 × 10^8^, SD ± 1 × 10^7^; and 1 × 10^8^, SD ± 2 × 10^6^ particles per mL, respectively. However, these differences were not statistically significant, suggesting that both materials are suitable for research on larger biological entities such as EVs and viruses. 

As a final test, we performed a fluorescent dye CellVue absorption test in single-channel devices by confocal microscopy (see Figure 2f,g). CellVue is often used in cell membrane staining for cell, EV, and virus interaction research critical in LOC for respiratory disease research. Figure 2h demonstrates that OSTE absorbs 6.63 times less CellVue dye than PDMS (normalized total CellVue fluorescence of PMDS is 2358.63 SEM ± 7.94, while OSTE had 355.33 SEM ± 4.45), suggesting that OSTE likely has less background noise for membrane labeling than PDMS or that this is an artifact from differences in light scattering since laser settings were the same.

### 3.2. Engineering and Cell Culturing on OSTE vs. PDMS LOAC Devices

The LOAC devices presented herein consist of two microfluidic chambers separated by a porous membrane. Before biology experiments, device fabrication and bonding parameters were thoroughly optimized. It was found that devices cured with a high-intensity UV light (such as mask aligner, with the intensity of 20–50 mW/cm^2^) tend to yield poorer bonding performance in the form of unbonded areas despite slight under-curing of OSTE. This likely can be attributed to triggering OSTE thermal curing step; therefore, all devices were fabricated utilizing a non-collimated UV-LED light source [35]. After the bonding process, devices were placed in a plasma asher to activate the surface before ECM coating steps and reduce the contact angle of OSTE and PC [46].

To evaluate the devices’ bonding performance, top and bottom channels were connected to a pressure system, and DI water was passed through the device at 1 mL/min in each of the channels. During the pressure testing, the device was carefully examined for leaking. The flow rate was chosen to be around two orders of magnitude higher than the operating flow rate serving as a safety factor. 

**Figure 3:**
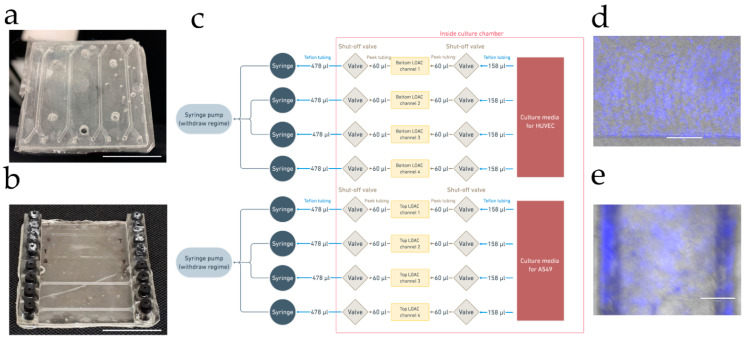
(**a**) Images of the PDMS LOAC device; white scale bar represents 25 mm. (**b**) Image of the OSTE LOAC device; white scale bar represents 25 mm. (**c**) Schematic diagram of the device cultivation system. (**d**) A549 cell monolayer on PDMS LOAC membrane after fixation labeled with DAPI on EVOS M5000 Imaging System (a combined image of transmitted and DAPI channels). (**e**) A549 cell monolayer on OSTE LOAC membrane after fixation labeled with DAPI on EVOS M5000 Imaging System (a combined image of transmitted and DAPI channels). Blue color represents DAPI-stained cell nucleus; white scale bar represents 300 µm.

Next, we compared LOACs microfabricated from OSTE and PDMS (see Figure 3a,b) for HUVEC (human umbilical vascular endothelial cells) and A549 (human lung carcinoma) cell coculturing on each side of the membrane by using the simple LOAC culturing protocol described in methods. PDMS, OSTE, and PC materials are already proven in numerous articles to be compatible with different cells growing on them [8,9,11,32,47,48,49,50,51,52]; therefore, we aimed to compare standard LOAC functionalization and application in immunofluorescence since there is a difference in light transmission and fluorescent dye absorption between polymers. HUVEC cells were used to represent endothelial cells on the bottom channel, while A549 cells were used as representative of lung epithelium on the top channel. Cells were cultured seven days on both devices without any leakage or bubble problem thanks to an in-house developed LOAC cultivation jig with shut-off valves for each channel at both entry and exit of the fluid paths (see Figure 3c). While the cell monolayer was monitored in the PDMS device, it was impossible to evaluate cell confluence of cells in the OSTE device with the EVOS M5000 Imaging System, probably due to decreased light transmission (see Figure 3d,e). 

**Figure 4:**
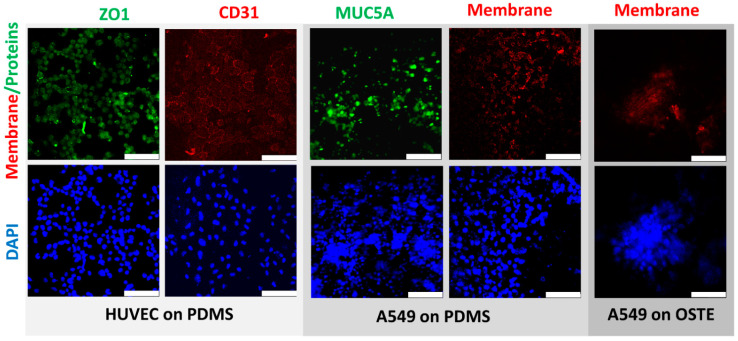
Comparison of confocal microscopy pictures of human umbilical vascular endothelial cells (HUVEC) and A549 cells between PDMS and OSTE LOACs. Blue color represents DAPI-stained cell nucleus. CD31 antibody stained with APC and CellVue are represented as red color. ZO1 and MUC5A stained with FITC are represented in green color. White scale bar represents 100 μm.

Further, we compared HUVEC and A549 cells between PDMS and OSTE LOACs by confocal microscopy (Figure 4). Cell nuclei were stained by DAPI, while CD31 (platelet endothelial cell adhesion molecule) antibody stained with APC was used as an endothelial cell-specific marker and antibody against tight junction marker ZO1 (Zonula occludens-1) stained with FITC was used as functional HUVEC monolayer marker. Results confirmed that HUVEC cells were growing in a monolayer on PDMS LOAC as expected based on CD31 and ZO1 markers. FITC-stained MUC5A antibody was used as a marker for lung epithelial cells since A549 cells produce this protein [53] that is human airway specific [54]. Based on Figure 4, results confirm that A549 also produces a monolayer on PC membrane in PDMS LOAC. CellVue was used as membrane dye for A549 cells and compared the CellVue background between PDMS and OSTE LOACs since there was a significant difference in absorption tests. While CellVue had some background noise that could be subtracted by image processing in PDMS LOAC, we could not obtain any good-quality pictures from OSTE LOAC with confocal microscopy. When imaging PDMS LOAC, DAPI signal was initiated with 1% of diode laser power, yet in OSTE LOAC, some barely specific fluorescence was detected only with 90–100% laser power. These results suggest that CellVue absorption differences are due to the poor light transmission and clearly demonstrate that OSTE polymer light transmission characteristics or LOAC production from OSTE need to be improved since LOAC microscopy is a crucial aspect of cell monitoring and evaluation of response to respiratory diseases and treatments.

**Figure 5:**
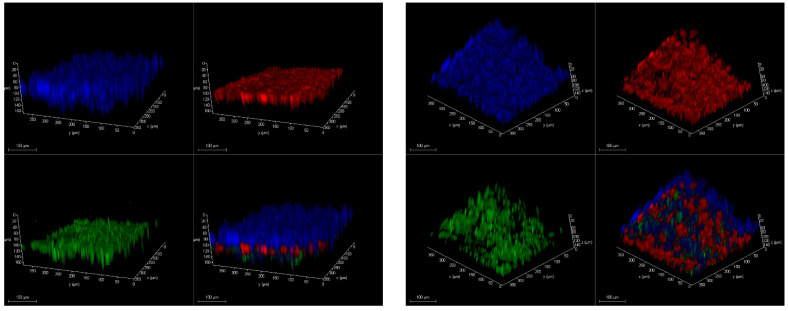
Confocal microscopy of 3D images of HUVEC and A549 cells on both sides of PC membrane in PDMS LOAC device from two different angles. Blue color represents DAPI-stained cell nucleus. The green color represents FITC-stained ZO1 antibody. The red color represents APC-stained CD31 antibody.

In Figure 5, we demonstrate functional monolayer formation on both sides of PC membrane in PDMS LOAC, where ZO1 (green) and CD31 (red) positive HUVEC cells are on the bottom channel and A549 cells are on the top channel. Due to the poor light transmission, it was impossible to acquire the same image set with confocal microscopy on OSTE LOAC. Therefore, we have not confirmed functional monolayer formation on both sides of the PC membrane within OSTE LOAC. However, based on previous literature of PC membrane [51,52,55] and OSTE polymer biocompatibility [32,47,48] and that some signal was detectable from DAPI and CellVue, this suggests that cells grow inside OSTE LOAC, but the quality of cell monolayer cannot be evaluated. These results confirm that there were no problems with the current cell growing protocol on PDMS and probably on OSTE LOAC and that the final conclusion about OSTE LOAC cell cocultivation on the membrane cannot be drawn due to the light transmission problems.

## 4. Discussion

One of the key reasons to look into alternative materials for LOAC and OOC fabrication is the manufacturability of the devices mentioned by the ORCHID roadmap [56]. PDMS microfluidic device fabrication has been refined over the years since the original publications of Whitesides, yet it remains slow due to the PDMS curing time [13,19,24]. The unscalable manufacturing process is an inherently limiting factor of truly widespread OOC device uptake [57]. Furthermore, in addition to the channel fabrication process being complex, the bonding protocol is also nontrivial. The use of commercially available track-etched membranes allows using a scalable membrane fabrication process. However, it also requires an elaborate surface modification to PDMS and membrane material to ensure covalent bonding, such as that seen in Figure 1 of this article, based on the protocol developed previously [40]. If PDMS membranes are used for this process, then the bonding process is significantly more straightforward (i.e., just plasma treatment [58]) and slower due to PDMS curing.

Microfluidic devices have been realized using various polymer materials [59]. Thermoplastics are often regarded as the ultimate material class for mass manufacturability due to the fact that a large number of materials are compatible with injection molding, capable of producing millions of units per annum [57]. As examples, it is worth mentioning polypropylene [60], polycarbonate [61], and polymethyl methacrylate (PMMA) [62]. Yet, this article has been focused on materials that can be prototyped in a typical academic setting, with access to a standard cleanroom environment, but can also be used to fabricate devices at larger volumes. Such material examples are OSTE, Flexdym [63], Styrene Ethylene Butylene Styrene (SEBS) [26], and polyurethane [64]. SEBS-based and polyurethane materials can be especially useful due to their flexible nature, which can be used in active element fabrication such as valves [64,65]. We have focused on OSTE material particularly due to its bonding properties. Contrary to PDMS, OSTE fabrication relies on UV curing and the emerging reaction injection molding process [66,67]. Furthermore, there have been examples of simple microfluidic structure reaction injection molding [35]. Although the shown parts do not exhibit the same complexity as a LOAC, it is a step towards mass manufacturability. For example, multichip curing in a single step exposure can be achieved using shadow masking of liquid runners between cavities, which removes the die-cutting step required in the PDMS fabrication process. Similarly, the OSTE bonding process is significantly simplified, owing to the available epoxy groups on the surface. The bonding process is then finished with a long-curing step, like in the PDMS process. In both processes, the temperature limiting factor is membrane material used in OOC devices [68]. Therefore, it is feasible to assume that OSTE could be utilized for the volume manufacturing process of LOAC devices through the use of reaction injection molding [67].

One of the main advantages of OOCs, including LOACs, is the several tissue level co-functionalities in fluid–air flow that is not possible currently for other in vitro models. Therefore, these models are attractive for preclinical tests for new drug testing or repurposing [10,23,69]. This is particularly attractive in multi-OOC systems where drug off-targets or synergy can be modulated. However, PDMS strongly absorbs small hydrophobic molecules, which is a significant drawback and requires extensive computational modeling in silico for drug pharmacokinetics and pharmacodynamics to recapitulate in vivo conditions. Moreover, new drug lead absorption in PDMS needs to be quantified before testing in OOC from PDMS, bringing additional variability in computational models [29]. In this study, we confirmed that OSTE polymer is a better-suited material than PDMS for drug testing and repurposing goals since it has several times less absorption of rhodamine B, which represents a small molecule drug in Figure 2b, which has also been published in several papers [31,32]. 

In this paper, to the best of our knowledge, we demonstrated for the first time that OSTE does not inhibit RNA isolation, cDNA synthesis, or PCR, which is critical for downstream analysis of cells in LOAC. According to previously published data, PDMS has one of the most negligible inhibitory effects on PCR among many polymers similar to polypropylene (PP) or polytetrafluoroethylene (PTFE) that are used in conventional tubes, plates, and microchips used for PCR [44]. According to our data represented in Figure 2c, OSTE has the same properties in PCR as PDMS, suggesting that it is suitable for downstream analysis in LOAC and OOC overall or another PCR-based lab on a chip device for on-point diagnostics, for example.

For the first time, we also demonstrated that OSTE does not significantly absorb EVs, which is similar to PDMS based on NTA data in Figure 2d. Commercial adipocyte-derived mesenchymal stem cell EVs were selected due to mesenchymal stem cell-derived EVs’ therapeutic effect, which could be applied in the future for drug transport and treatment of different diseases [70]. Since EVs are involved in almost all physiological processes, including pathophysiology and recovery of lungs from respiratory diseases, including virus infection, they are promising candidates for future treatments, including vaccination [71]. Therefore, LOACs and OCCs overall or lab on a chip devices from OSTE could be potentially applicable for drug-loaded EV testing or isolation/production.

However, microscopy remains a critical LOAC and OOC evaluation aspect, and many of the experimental results are dependent on reflective/transmissive light microscopy and confocal microscopy [72,73]. A notable drawback of OSTE material is the optical characteristics. The most glaring difference between PDMS and OSTE is the total transmittance, as seen in Figure 2a, which has been reported previously, especially for OSTE 322 that has substantial absorption below 380 and 420 nm [35]. This is significant because DAPI is a widely used fluorescent molecule with an excitation peak of 358 nm [74]. Furthermore, the nature of the loss of transmission is essential—previously, it has been noted that the loss occurs from the light scattering properties of OSTE rather than just pure absorption [32]. The scattering is also evident in the confocal microscopy images presented here, which lack the crisp image quality and detail seen in PDMS devices. Moreover, some reports have mentioned that heat treatment could affect OSTE optical properties [75]; however, here the thermal treatment of OSTE LOAC was critical for bonding of OSTE and PC membrane. As a summary, see Table 1 for a comparison between PDMS and OSTE for various properties important for LOAC development.

To capitalize on the improved material characteristics, further study would be necessary for understanding the light scattering cause in the material. An immediate research direction would be a thorough study of UV polymerization wavelength and intensity effect on OSTE transmission. Furthermore, given that there are two components in the OSTE mixture, the effect of material separation in time (for example, during the degassing step) must be evaluated. Further, the effect of thermal treatment must be carefully examined with the intent to evaluate if the temperature profile and absolute curing temperature have any effect on the scattering properties.

## 5. Conclusions

In conclusion, this paper reports the use of OSTE materials for LOAC fabrication and contrasts it with conventional PDMS LOAC devices. As discussed in the respective sections, OSTE materials have clear advantages over PDMS in terms of device fabrication, large volume scalability, and small molecule absorption. However, current material optical characteristics impede widespread adoption of the OSTE materials for OOC, especially given the crucial contribution of confocal microscopy in OOC research. Therefore, for unlocking the true OSTE material potential, material transmission properties must be improved, emphasizing reducing the light scattering of the material.

## Figures and Tables

**Table 1:** A comparison between polydimethylsiloxane (PDMS) and off-stoichiometry thiol–ene (OSTE) properties.

Property	PDMS	OSTE
Light transmission in the 300–800 nm range	Good, comparable to glass	Intermediate, multilayer chips suffer from significant light scattering
Cell viability	Good cell viability [23]	Acceptable cell viability [48]
Bonding to PC membrane	Intermediate steps necessary to functionalize PDMS and membrane materials [40]	Readily bondable via epoxy groups available prior to thermal treatment [47]
Surface modification	Intermediate steps necessary [76]	Readily available –OH or –SH groups [31]
Stability in chloroform and ethanol, solvents used for cell fixing	Medium–poor [77]	Good [75]
Gas permeability	High [78]	Low [36]
